# Effectiveness of Annealing Blocking Primers versus Restriction Enzymes for Characterization of Generalist Diets: Unexpected Prey Revealed in the Gut Contents of Two Coral Reef Fish Species

**DOI:** 10.1371/journal.pone.0058076

**Published:** 2013-04-08

**Authors:** Matthieu Leray, Natalia Agudelo, Suzanne C. Mills, Christopher P. Meyer

**Affiliations:** 1 Laboratoire d'Excellence “Corail”, USR 3278 CRIOBE CNRS-EPHE, CBETM de l′Université de Perpignan, Perpignan, France; 2 Department of Invertebrate Zoology, National Museum of Natural History, Smithsonian Institution, Washington, D.C., United States of America; University of Veterinary Medicine Hanover, Germany

## Abstract

Characterization of predator-prey interactions is challenging as researchers have to rely on indirect methods that can be costly, biased and too imprecise to elucidate the complexity of food webs. DNA amplification and sequencing techniques of gut and fecal contents are promising approaches, but their success largely depends on the ability to amplify the taxonomic array of prey consumed and then match prey amplicons with reference sequences. When little *a priori* information on diet is available or a generalist predator is targeted, versatile primer sets (also referred to as universal or general primers) as opposed to group- or species-specific primer sets are the most powerful to unveil the full range of prey consumed. However, versatile primers are likely to preferentially amplify the predominant, less degraded predator DNA if no manipulation is performed to exclude this confounding DNA template. In this study we compare two approaches that eliminate the confounding predator template: restriction digestion and the use of annealing blocking primers. First, we use a preliminary DNA barcode library provided by the Moorea BIOCODE project to 1) evaluate the cutting frequency of commercially available restriction enzymes and 2) design predator specific annealing blocking primers. We then compare the performance of the two predator removal strategies for the detection of prey templates using two versatile primer sets from the gut contents of two generalist coral reef fish species sampled in Moorea. Our study demonstrates that blocking primers should be preferentially used over restriction digestion for predator DNA removal as they recover greater prey diversity. We also emphasize that a combination of versatile primers may be required to best represent the breadth of a generalist's diet.

## Background

Molecular analysis of gut or feces contents using polymerase chain reaction-based techniques (PCR) has the potential to characterize predator diets and unveil the complexity of food webs [1]–[3]. Prey-specific DNA fragments can be detected from unidentifiable prey items, even after several hours of digestion [4]–[6]. Amplified sequences are then compared against existing prey sequence databases to provide a qualitative dietary analysis of any vertebrate or invertebrate predator [7].

The success of molecular analyses of animal gut contents primarily depends on the ability to detect the range of prey consumed via PCR amplification. Gut contents may contain undigested (recently consumed) or indigestible individual prey items which can be individually dissected and from which a target gene can be amplified and sequenced to supplement morphological identification [8]–[10]. However, for numerous predators that feed either upon soft bodied prey, by liquid ingestion (i.e. invertebrates) or consume small items that are rapidly digested, most dietary information will be obtained from the semi-digested tissue homogenate (a mixture of DNA templates) (e.g. [11]). This homogenate will contain DNA traces from a large pool of prey consumed [12]. However, semi-digested prey homogenates commonly contain small amounts of highly degraded prey DNA [7] mixed with the prevalent high quality DNA of the predator itself [13]. Predator DNA co-amplification will often prevent or bias prey recovery if no preventive measures are taken [14]–[21].

One method commonly used to exclude predator DNA is the design of species- or group-specific primers that target one or a group of prey species of interest without binding to predator DNA [7], [22], [23]. These primers are particularly useful when either predators have a diet restricted to one or a few taxonomic groups (i.e. bats feeding on insects [14], [24], [25]), or the aim of the study is to detect specific food items (i.e. detect consumption of invasive species by generalist predators [15], [16]). On the other hand, using multiple sets of species or group-specific primers to screen the gut or feces contents of generalist predators for food web studies will be costly, time consuming and might produce false negatives as sequence datasets used to design primers are often incomplete [17]. In this case, versatile PCR primer sets (also referred to as universal or general primers), designed to bind to a highly conserved region across taxa, will be more powerful to establish an exhaustive list of prey consumed.

However, as versatile primers might also have affinities with the DNA of the predator itself, they often need to be used in combination with a predator removal procedure (reviewed in [21]). One technique is to cleave and exclude predator DNA prior to and after PCR amplification using a restriction enzyme [18], [26]. The technique was first implemented by Blankenship and Yayanos (2005) [18] who managed to characterize the broad diversity of food items consumed by deep sea scavenging amphipods and a bivalve species. A second competing strategy for predator DNA subtraction was developed by Vestheim and Jarman (2008) [20] who managed to completely remove predator sequences using predator specific annealing blocking primers in order to analyze the diet of Antarctic krill by targeting short ribosomal prey fragments. Blocking primers are modified primers which overlap with one versatile primer binding site and extend into a predator specific sequence. They help prevent predator DNA amplification but simultaneously enable amplification of DNA from prey items [19].

Although the possibilities offered by PCR based diet analyses have been improved, the use of these predator DNA removal strategies has raised concerns about potential prey DNA cleavage or blocking which have so far not been evaluated [2], [18], [20], [21], [26]. As enzyme recognition sites may often be shared among predator and prey items, the appropriate enzyme must be carefully selected to minimize the loss of target templates [18], [26]. Similarly, the specificity of annealing blocking primers to predator DNA is determined by both the amount of variability across the target DNA fragments of potential prey and the extent to which the blocking primer will extend into the predator specific sequence. Blocking primers must have their 3′end located in a highly variable region to maximize the probability of a mismatch between the predator specific blocking primer and the prey it consumed [2], [19], [20].

This study aims to compare the performance of these two predator DNA removal strategies in the detection of prey templates from the semi-digested prey homogenate from the gut contents of two coral reef associated hawkfish species using two versatile primer sets targeting the Cytochrome c. Oxidase subunit I (COI) region. The arc-eye hawkfish *Paracirrhites arcatus* has a distribution which extends from the central Pacific to the Eastern coast of Africa. It occurs in the lagoon and on reef slopes and uses *Pocillopora* coral heads as a preferred habitat [27]. Its diet, determined from morphological identification of prey hard parts, is thought to be mainly composed of decapods and fish [28]. The flame hawkfish *Neocirrhites armatus* is a highly prized aquarium fish commonly occurring at shallow depths on reef fronts. The species is strictly associated to corals of the genus *Pocillopora* and *Stylophora* [29] and has been reported to feed upon motile invertebrates, but information about its feeding habits is scarce [28]. Specimens of both predatory fish species were collected from the island of Moorea (French Polynesia) which has been the host of the “Moorea BIOCODE” project (http://www.biocode.berkeley.edu/), an all-taxa biodiversity inventory, whose goal is to provide a library of genetic markers for all non-microbial terrestrial and marine species of the tropical island ecosystem [30]. At the time of fish collection and analyses, the sequence library contained DNA signatures (COI) for ∼1500 reef associated vertebrate and invertebrate species upon which fish may potentially feed. This preliminary COI database was used to (1) provide guidelines for the choice of a restriction enzyme and the design of predator specific blocking primers that would minimize loss of prey (false negatives), and (2) identify prey to provide a preliminary assessment of the feeding ecology of two predatory fish species. We believe that the methodological guidelines presented in this study are applicable for dietary analyses of any generalist predator and will ultimately encourage further research into food web dynamics in both marine and terrestrial ecosystems.

## Materials and Methods

### Hawkfish collection and gut content DNA extraction

Ten specimens of the arc-eye hawkfish *Paracirrhites arcatus* and ten specimens of the flame hawkfish *Neocirrhites armatus* were spearfished by M. Leray at sunset on the outer reef and the lagoon of the North shore of Moorea, French Polynesia (17°30′S, 149°50′W), during the Austral Winter 2009. Fish were individually preserved in cold 50% ethanol *in situ*. The digestive system was then dissected within 3 hours and preserved in eppendorf tubes containing 80% ethanol. Following storage at −20°C for up to three months, the stomach and intestine were carefully opened and undigested distinguishable prey items in the stomach, as well as indigestible remains in the intestine, such as gastropod shells, were carefully removed, rinsed with distilled water and placed in individual annotated tubes containing 80% ethanol. Total genomic DNA was extracted from individual prey by means of an automated phenol chloroform extraction with the Autogenprep 965 (Autogen, Holliston, MA) using the mouse tail tissue protocol with a final elution volume of 100 µL. Mollusc shells were broken to access potential tissue. The remaining mixture of semi-digested prey was used for total genomic DNA extraction with QIAGEN DNeasy Blood & Tissue individual columns. As mixed genomic DNA contained PCR inhibitors, each sample was then cleaned using the PowerClean DNA clean-up kit (MO BIO). Approval was granted from our institutional animal ethics committee, le Centre National de la Recherche Scientifique (CNRS), for sacrificing and subsequently dissecting fish (Permit Number: 006725). None of the fish species are on the endangered species list and no specific authorization was required from the French Polynesian government for collection. When possible, we made an attempt to spear the fish behind the gills.

### Morphological and DNA based identification of undigested and indigestible prey items

**Figure 1:**
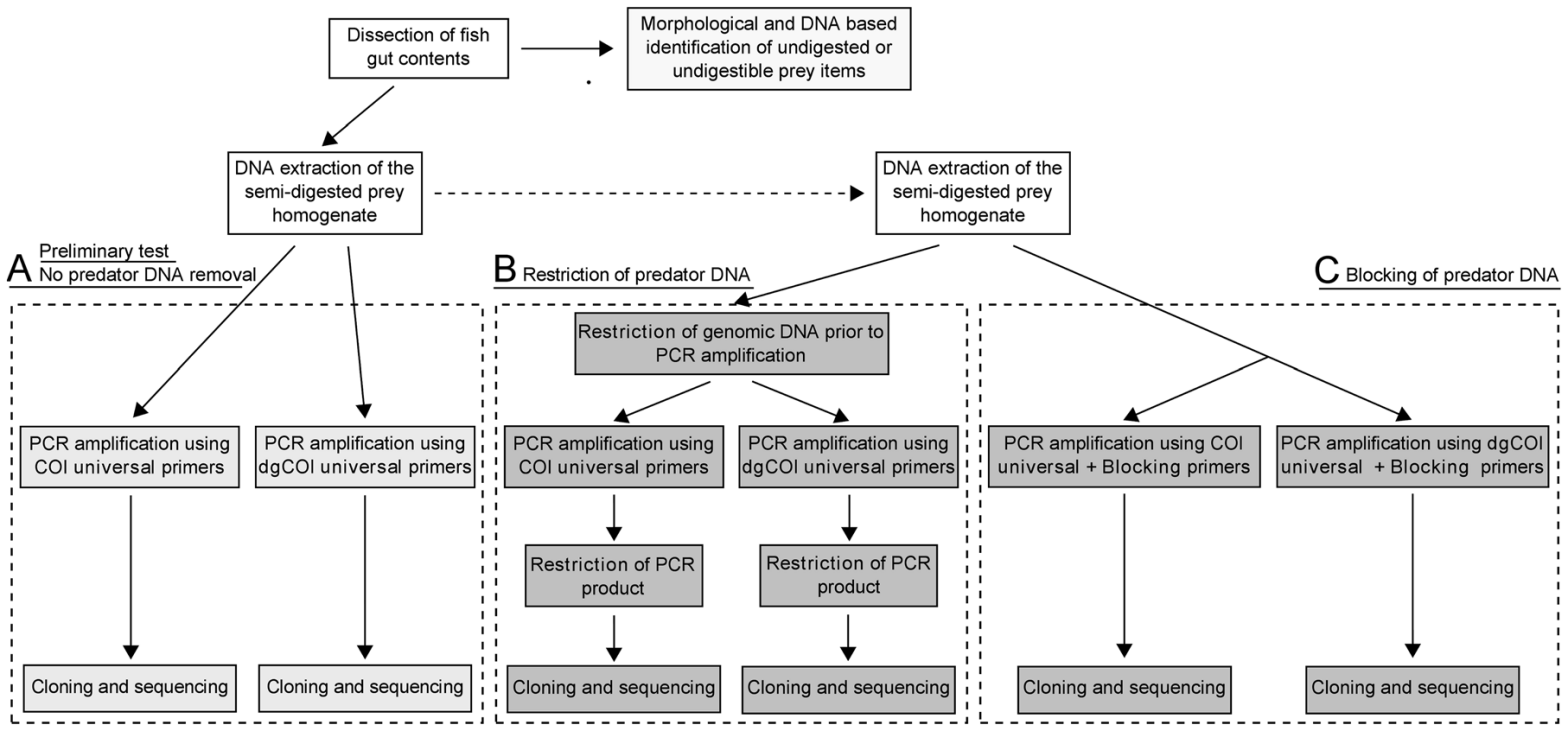
Overview of the protocol used for PCR based dietary analyses. The versatile primer set “COI” [33] and its degenerate version “dgCOI” [34] were used. The dashed arrow indicates that an aliquot of the semi-digested prey homogenate DNA extract was used to evaluate the performance of predator DNA removal techniques (B: restriction enzymes; C: annealing blocking primer).

Following morphological identification to the lowest taxon level, DNA was extracted from these large residual elements and PCR amplifications of the COI were performed (Fig. 1) as 20 μl reactions with 0.6 μl of 10 µM of each versatile forward and reverse primer [31], 0.2 μl of Biolase *taq* polymerase (Bioline) 5U.μl^−1^, 0.8 μl of 50 mM Mg^2+^, 1 μl of 10 µM dNTP and 1 μl of genomic DNA. PCR thermal cycling conditions were: 5 min at 95°C; 35 cycles of 30 s at 95°C; 30 s at 48°C; 45s at 72°C; and a final 5 min at 72°C. Sequences were generated in both directions.

### Preliminary evaluation of predator DNA co-amplification from semi-digested prey homogenates

We amplified COI from the gut contents of four of the ten *N. armatus* and four of the ten *P. arcatus* without using any predator DNA removal strategy (Fig. 1A). PCR amplifications were performed for each sample using 2 μl of digested genomic DNA and two sets of versatile COI primers commonly used for invertebrate species barcoding (1) “COI”: LCO1490: GGT CAA CAA ATC ATA AAG ATA TTG G; HCO2198:TAA ACT TCA GGG TGA CCA AAA AAT CA [31]; (2) “dgCOI”: dgLCO1490: GGT CAA CAA ATC ATA AAG AYA TYG G; dgHCO2198:TAA ACT TCA GGG TGA CCA AAR AAY CA [32]. The previously described PCR cycling profile was used. Each PCR product was cloned using the TOPO TA cloning kit (Invitrogen). 30 clones per sample were directly amplified with M13 primers and sequenced in one direction using the T3 primer following the manufacturer's instructions.

### Guidelines for developing two predator DNA removal techniques

#### Restriction enzymes

We evaluated the cutting frequency of commercially available enzymes among taxa, by mapping the presence of restriction sites within the COI barcoding region of three diverse groups found within the Moorea reef ecosystem that are represented in the BIOCODE sequence database: ≈330 decapod morpho-species (947 sequences), ≈380 ray-finned fish morpho-species (758 sequences) and ≈170 gastropod morpho-species (271 sequences) using the program Cleaver [30]. We provide approximate numbers of morpho-species in each group because a number of specimens remain unidentified to the species level or belong to undescribed species [8]. Enzymes with an effective recognition sequence length ranging between 6 and 15 base pairs which include either specified base pairs only (i.e. EcoRI: GAATTC) or degenerate sites (i.e. GdiII: CGGCCR) were mapped ( Table S1). Restriction enzymes that optimally remove predator DNA from the gut content homogenate while minimizing digestion of non-predator targets were chosen.

#### Annealing blocking primers

COI fragments of species belonging to ray-finned fish, decapods and gastropods were aligned and the information content (entropy, hx) at each position of the 3′ end of the COI fragments was calculated using Bioedit [33]. Entropy plots illustrate the level of variability at each position in an alignment of sequences. At conserved sites (e.g. “C” in all sequences) entropy is 0. If all four nucleotides occur at a position with a frequency of 0.25, then the entropy value is maximal. A suitable binding site was then identified to maximize the probability of a mismatch between the 3′ end of the two predator specific annealing blocking primers and sequences of potential prey. Predator specific annealing blocking primers overlapping with the reverse COI versatile primer site were designed for *P. arcatus* and *N. armatus*.

Moorea BIOCODE specimen lists and photographs used in this study are available on http://www.biocode.berkeley.edu/ and COI sequences are public on the Barcode of Life Data Systems (BOLD) www.boldsystems.org [8] (project MBMIA for decapods, project MBMIB for gastropods and project MBFA for fish).

### Comparison of predator DNA removal strategies for prey detection

We compared the efficiency of the predator removal methods for the detection of prey when using the two sets of versatile primers (Fig. 1B and C). Aliquots of the ten specimens of each species of fish were individually amplified with each of the following treatments: (1) Pre- and post-PCR predator DNA restriction and “COI” primer set, (2) Predator specific annealing blocking primer and “COI” primer set, (3) Pre- and post-PCR predator DNA restriction and “dgCOI” primer set, (4) Predator specific annealing blocking primer and “dgCOI” primer set. As the “COI” primer set fails to amplify *N. armatus*, DNA removal methods were unnecessary for this predator. Laboratory protocols are detailed below.

#### Restriction enzymes

First, a restriction digestion of the genomic DNA was performed for each sample prior to PCR amplification. For each gut sample, 25 μl of DNA (4–20 ng/μl) was used in a total reaction volume of 40 μl containing 4 units of enzyme and incubated for 16 hours until a final heat inactivation. Three PCR reactions per sample were performed to account for potential PCR drift [34]. PCR products obtained with both sets of primers were further digested for 12 hours in a total reaction volume of 15 μl containing 4 μl of PCR product and 1.5 units of enzyme before a final heat inactivation. We used a positive control for all restriction digestion reactions. Digested PCR products were run on a 1.5% agarose gel and the ∼650 bp COI fragments excised. DNA fragments were purified from the gel slice using gelase (Epicenter Biotechnologies). The PCR product obtained from the gut contents of two specimens was cloned separately to give an indication of prey diversity. Then, PCR products were paired according to COI primer sets and DNA concentration (measured by Qubit® Fluoremetric quantification) before cloning. We sequenced 35–40 clones per sample, as we assumed this was sufficient to describe the most commonly consumed prey items by each specimen.

#### Annealing blocking primers

The blocking primer was included at 10 times the concentration of COI versatile primers during amplification [20]. Each sample was amplified with both sets of versatile COI primers. COI fragments were similarly gel excised, purified, cloned and sequenced to enable data comparison between the sets of COI amplicons obtained after the removal of predator DNA using restriction enzymes.

We examined differences in the detection of prey template (number of species amplified) between treatments. For prey detection in the gut contents of *P. arcatus*, a repeated measures ANOVA design was used to examine the main effect of predator removal, the main effect of primer set and the interaction between the two factors. Paired t-tests were then computed to determine the directionality of the effects and interpret the interaction. Since no predator DNA removal method was needed for prey amplification in the gut contents of *N. armatus* with the “COI” primer set, we only tested whether the mean number of prey detected using the “dgCOI” primer sets was higher using the blocking primer than the restriction enzyme. Count data (number of species) were square root transformed in order to meet parametric assumptions.

### Sequence dissimilarity threshold for prey species delineation

The evaluation of taxonomic diversity from clone libraries obtained from gut contents requires clustering sequences into Operational Taxonomic Units (OTU). Each OTU is then assumed to represent an evolutionary distinct lineage. We used COI sequence datasets from the Moorea BIOCODE project to determine the optimal sequence dissimilarity threshold for species delineation among decapods, ray-finned fish and gastropods. Uncorrected pairwise genetic distances were calculated between aligned sequences and the furthest neighbor algorithm, with 1% to 15% dissimilarity thresholds, was employed for sequence clustering. We ran a step function analysis (as in [35]–[37]) to determine the threshold upon which the number of OTUs becomes stable: that is the threshold where intra- and inter-specific variability do not affect diversity estimations. Sequence processing was implemented in Mothur [38].

### Taxonomic assignment of prey

Sequences obtained from clone libraries were edited, trimmed and translated into amino acids for alignment using ClustalW [39] implemented in Geneious Pro 5.0.3 [40]. Sequences with anomalies (stops or frame shifts) in amino acid translation were considered non-functional mitochondrial COI (e.g. pseudogenes) and removed. Chimeric sequences were identified using the program Bellerophon [41] and discarded. In order to cluster remaining sequences into OTUs, pairwise genetic distances were calculated and sequences were clustered in OTUs as previously detailed. To facilitate data handling, a single sequence per OTU exhibiting a minimum distance from the other sequences was chosen. “Representative sequences” were then identified based on their similarity to GenBank and BIOCODE sequence libraries using BLAST [42] searches implemented in Geneious [40]. Sequences matching bacteria were removed from the prey dataset. The remaining COI sequences could then be assigned to species if the BLASTN similarity was ≥98% [43]. Whenever species level assignment could not be achieved, we used the Statistical Assignment Package (SAP [44]) which uses a Bayesian approach to calculate the probability that a sequence belongs to a higher taxonomic group to that represented in reference databases. We used a posterior probability above 0.95 to confidently assign a sequence to a taxonomic group.

We tested whether the number of fish collected was sufficient to describe the diet of the two predator species. Expected species accumulation curves with 95% confidence intervals were computed using the program EstimateS [45], [46].

## Results

### Identification of undigested and indigestible items

Dissection of the digestive system of ten *P. arcatus* revealed very few visually distinguishable prey items. Only two nearly undigested specimens of the decapods *Trapezia tigrina* and *Galathea mauritiana*, as well as appendages of two brachyuran, one anomoura and one caridean shrimp were found in the stomach of five different fish. A gastropod shell (*Homalopoma)* was also recovered from one intestine. Even fewer items were gathered from the ten *N. armatus* digestive tracts: a chelae of a brachyura in one stomach, as well as a bivalve (juvenile Pectinidae) and a gastropod (Rissoidae) from one intestine.

COI amplification and sequencing of DNA extracted from decapod appendages enabled molecular identification to the species-level. A BLAST search in the BIOCODE database provided species level identifications (>98% similarity) for the brachyurans: *Pilodius flavus*, *Jonesius sp*. and *Liocarpilodes sp*. (chelae in *N. armatus*), the anomoura: *Galathea sp.* and the caridean shrimp: *Alpheus dolerus*. DNA extracted from the three shelled mollusks found at the terminal section of the fish intestines (close to anus) was at a very low concentration (<1ng) and PCR amplifications were not successful. At that point, the gastropod shells could have been empty. Small gastropod shells can also be inhabited by hermit crabs, so caution should be made when only identifying shells.

### Prevalence of enzyme restriction sites in BIOCODE COI sequence libraries

There was considerable variation in the cutting frequency of enzymes (Table S1). This was expected as enzymes vary greatly in the length of their recognition site and sequence-specificity. The vast majority (68%) of commercially available enzymes mapped have a 6 bp recognition site, with a cutting frequency ranging from 0 to 91% across all sequences (mean ± SE  = 15.37%±21.7). However, the prevalence of restriction sites of 6 bp enzymes without a degenerate site was lower (0 to 75%; mean ± SE  = 9.47±12.9). There was a small amount of variation in the enzyme cutting frequencies between decapod, ray-finned fish and gastropod sequences. Among the restriction enzymes cutting the *P. arcatus* COI sequence, BstXI (CCANNNNNNTGG) was the most absent across decapod, ray-finned fish and gastropod COI sequences (absent in 96%, 98%, 92% of sequences respectively). Similarly, BspEI (TCCGGA) was selected to restrict *N. armatus* DNA (absent in 98%, 91%, 93% of sequences respectively).

### Levels of variability across aligned COI sequences assessed for designing blocking primers

**Figure 2:**
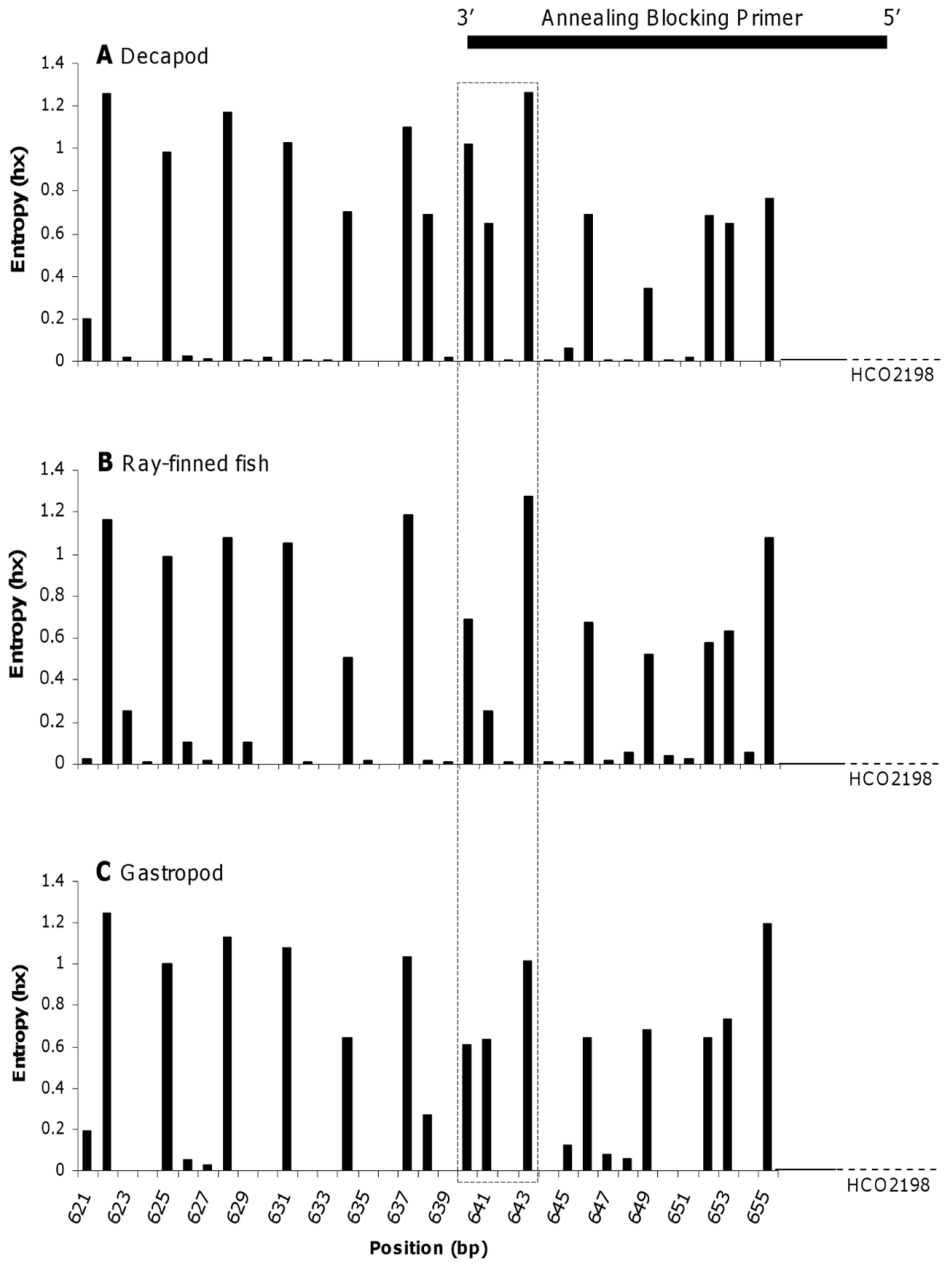
Variability at each position of the COI 3′ end region for the design of annealing blocking primers. Entropy plots were performed using 947 decapod COI sequences (A) (≈330 species), 758 fish COI sequences (B) (≈380 species) and 271 gastropod COI sequences (C) (≈170 species) collected and sequenced by the Moorea BIOCODE project. The black line shows the position where predator specific annealing blocking primers were designed for the two hawkfish species in order to minimize the probability of blocking prey amplification. The black box displays a region of high sequence variability across taxa making it a good-quality binding site for the 3′end of predator specific annealing blocking primers.

The 3′end of the COI region shows considerable levels of variation across decapod, ray-finned fish and gastropod sequence alignments (Fig. 2) with the most highly variable sites found at third-position nucleotides. High entropy values between the positions 640 and 643 (see dashed box in Fig. 2) make this region a potential binding site for the 3′end of predator-specific annealing blocking primers. As both hawkfish species sequences are TCTT at these four sites, the probability of having at least one mismatch between decapod, fish or gastropod templates and hawkfish DNA sequence will be equal to 89%, 76% and >99% respectively (1– P(T) × P(C) × P(T) × P(T), see frequency values in Table 1). Furthermore, designing annealing blocking primers with the 3′ end sitting at the position 640 keeps the primers short (<30 bp) and TMs low for effective annealing [47].

**Table 1:** Frequency (%) of each nucleotide for the alignment of 947 decapod (D), 758 fish (F) and 271 gastropod (G) COI sequences at the binding site (positions 640 to 643) of the 3′end of predator specific annealing blocking primers designed in this study.

	%A			%C			%T			%G		
Position (bp)	D	F	G	D	F	G	D	F	G	D	F	G
640	12.7	0.2	3.3	24.9	40.3	15.1	59.5	59.5	0.7	2.9	0	80.8
641	0	0	0	65.5	92.9	33.6	34.5	7.1	66.4	0	0	33.6
642	0	0	0	0.1	0.1	0	99.9	99.9	100	0	0	0
643	43.7	17.1	58.7	17.6	26	6.3	28	44.2	5.9	10.8	12.7	29.2

Prey template-primer mismatch will minimize the probability of blocking prey COI amplification.

Blocking primers (5′–3′) were designed as CAAAGAATCAAAACAGGTGTTGATAAAGA and CAAAGAATCAGAACAGATGTTGGTAAAGA for *N. armatus* and *P. arcatus* respectively, with the last 10 base pairs overlapping with the reverse versatile primer binding site. In order to prevent elongation without affecting their annealing properties, primers were modified at the 3′end with a Spacer C3 CPG (3 hydrocarbons) [20].

### Performance of predator removal strategies for the detection of prey templates from semi-digested prey homogenate

Preliminary tests indicated that predator DNA sequences were largely predominant in clone libraries when amplifying *P. arcatus* gut contents using both versatile COI primers sets without any predator DNA removal method (Fig. 1A) (mean ± SE: with “COI”  = 94.1±0.8%, n = 4; with “dgCOI”  = 98.3±1%, n = 4) and *N. armatus* when using the “dgCOI” primer set only (mean ± SE: 86.7±2.7%, n = 4). On the other hand, *N. armatus* amplicons were absent from clone libraries when using the “COI” primer set which yielded only non-predator targets (Table 2).

**Table 2:** Summary of results from the experimental evaluation of predator DNA removal techniques for the PCR amplification of the semi-digested prey homogenate in the gut contents of *Neocirrhites armatus* (n = 10) and *Paracirrhites arcatus* (n = 10).

	*Paracirrhites arcatus*				*Neocirrhites armatus*		
DNA removal	ENZYME		BLOCKING		NONE	ENZYME	BLOCKING
Primer set	COI	dgCOI	COI	dgCOI	COI	dgCOI	dgCOI
Number of clones sequenced	342	326	323	334	332	365	368
% Prey sequences	82	60	86	55	78	68	76
% Bacteria sequences	14	34	12	42	20	25	17
% Pseudogenes sequences	4	6	2	3	2	7	7
Number of prey OTUs (5–10%)	11	10	19	11	19	18	26

Two versatile primer sets (“COI”, Folmer et al., 1994; and “dgCOI”, Meyer, 2003) and two predator DNA removal strategies (restriction enzymes and blocking primers) were used. As the primer set “COI” (Folmer et al., 1994) does not enable amplification of the COI region of *N. armatus*, no predator DNA removal was required (see results of preliminary tests for host DNA co-amplification).

Both restriction enzymes and blocking primers were highly efficient at excluding predator DNA. Using the restriction enzyme procedure or adding a blocking primer at ten times the concentration of versatile primers led to negligible proportions of predator sequences (mean proportion of predator sequences <1%). Consequently, a total of 2390 clones were successfully sequenced from semi-digested prey homogenates of both fish species to compare the performance of predator DNA removal strategies for the detection of prey.

Prey sequences represented 55 to 86% of the total number of sequences in the unfiltered dataset for the different treatments (Table 2). Among the sequences extraneous to predator dietary analysis, bacterial genes accounted for between 12 to 42% of the total number of sequences. Pseudogenes were not as dominant in the clone libraries (2 to 7%) and chimeric sequences were rare (<0.01% – not reported in Table 2).

**Figure 3:**
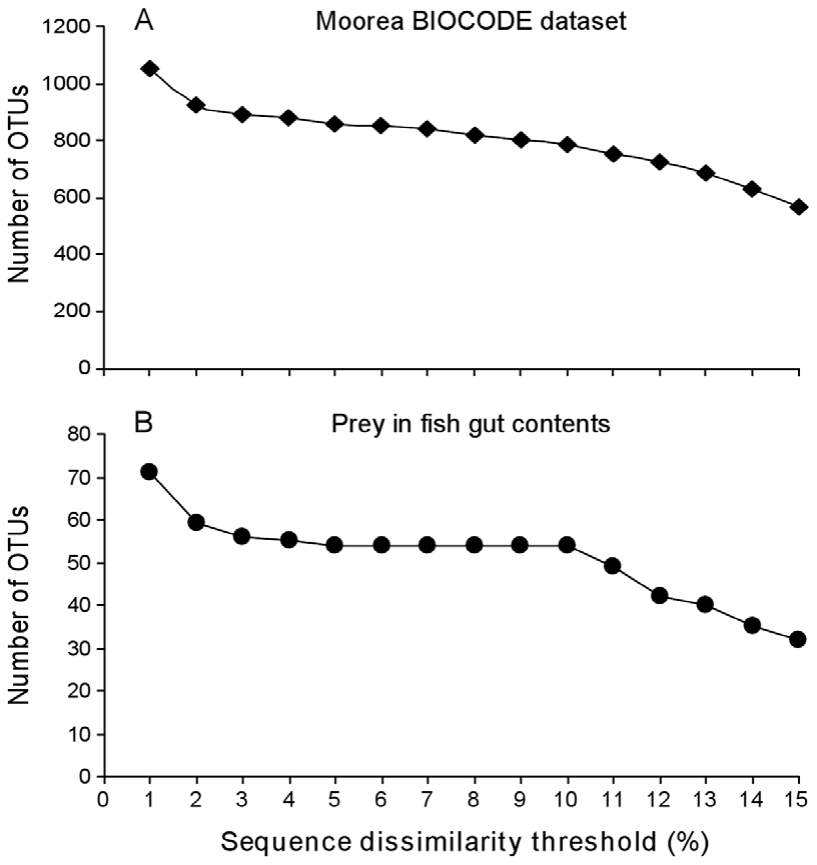
Evaluation of COI sequence dissimilarity thresholds for clustering sequences in Operational Taxonomic Units. We used 1976 COI sequences (∼880 morpho-species belonging to fish, decapods and gastropods) provided by the Moorea BIOCODE project (A) and the sequences amplified (clone libraries) from hawkfish gut contents (B).

**Figure 4:**
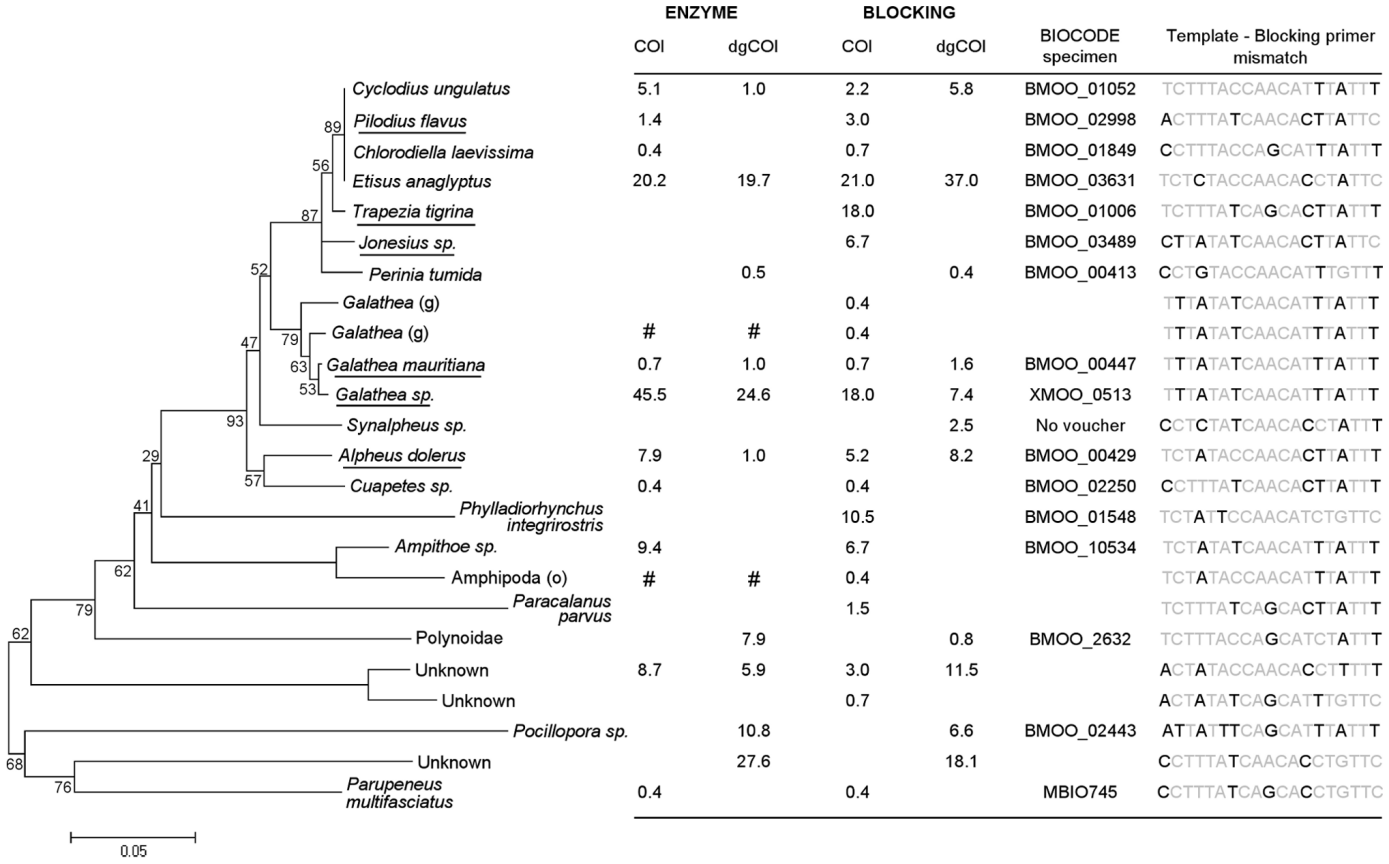
Prey items obtained from the gut contents of 10 specimens of *Paracirrhites arcatus*. One representative sequence per Operational Taxonomic Unit (OTU) (cutoff threshold  = 5–10%) was chosen to compute the neighbor joining (poisson corrected distance) of COI amino acid sequences using Mega [69]. Branch support was evaluated using 1000 bootstraps. The overall proportion of sequences for each OTU for each combination of versatile primer sets (“COI” and “dgCOI”) and predator DNA removal strategies used (Restriction enzymes and Blocking primers) are presented in the adjacent table. The name of the taxon is underlined when it was also identified using DNA barcoding of undigested remains. The BIOCODE number of the reference specimen to which an OTU match is given when the BLASTN similarity was greater than 98%. Pictures and sampling details of each reference specimen are available at http://mooreabiocode.org/. Whenever species level identification could not be achieved, we indicate the higher taxonomic group to which each prey sequence belongs (statistical assignment package – see methods). If not assigned to species-level, taxonomic rank is shown within parentheses (p: phylum, c: class; o: order, f: family, g: genus). Mismatches between the binding site of the predator specific blocking primer and amplified templates (positions 640 to 658 at the 3′end of the COI region) are presented. Prey sequences which were likely excluded during restriction digestion steps are marked # in the table.

**Figure 5:**
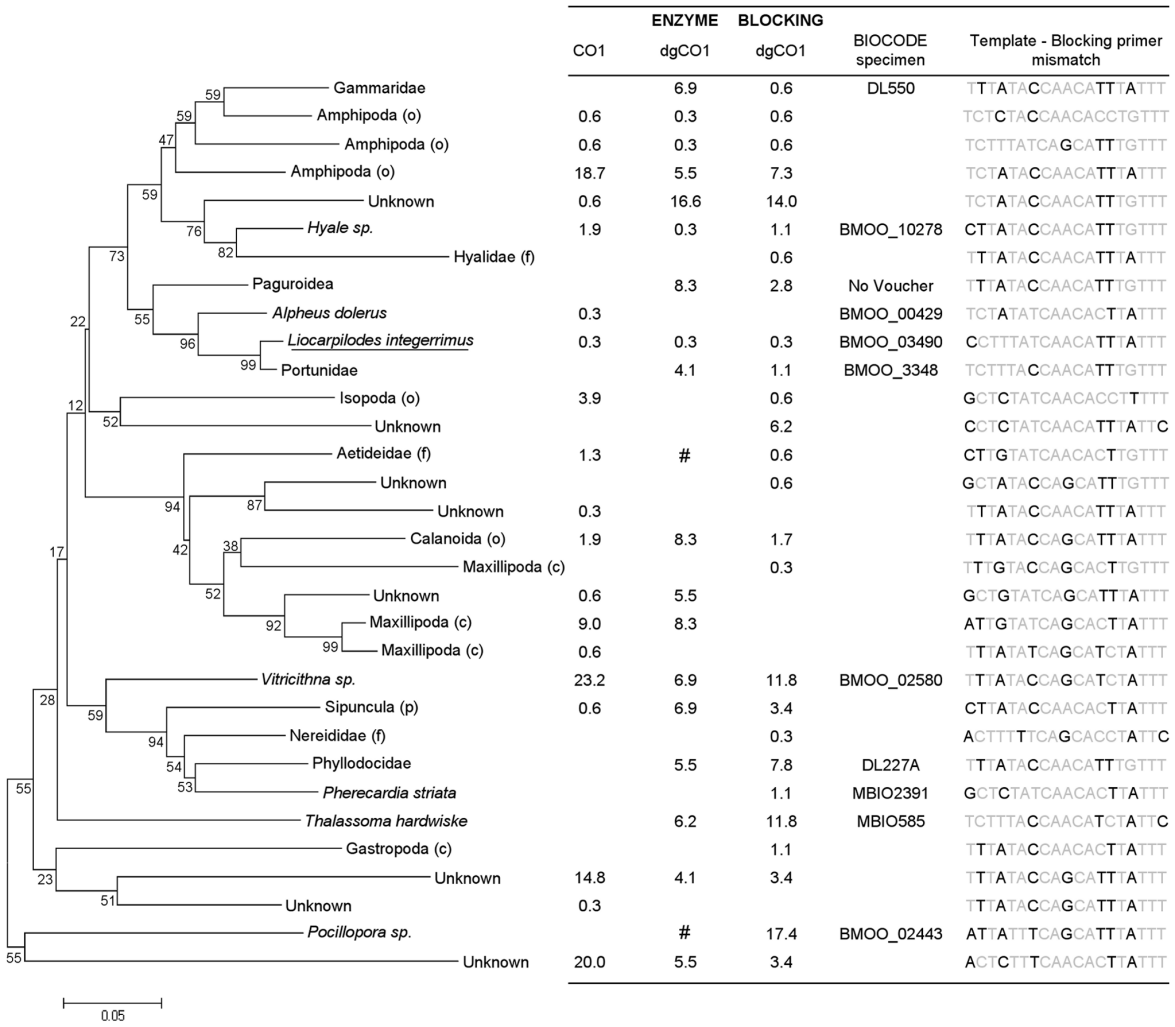
Prey items obtained from the gut contents of 10 specimens of *Neocirrhites armatus*. See legend of Figure 5 for further details.

Using BIOCODE sequence libraries, we found that there was steep decrease in the number of OTUs from 1% to 3% before reaching an inflexion point at 4% (Fig. 3A) which corresponds to a limit where intra-specific variation becomes negligible. The curve stabilizes from 4 to 10% before decreasing more sharply. There was a more distinct pattern for prey sequences recovered from hawkfish gut contents (clone libraries) where the total number of OTUs was constant from 5% to 10% sequence dissimilarity (Fig. 3B). Within this range, 24 and 32 prey OTUs were found in *P. arcatus* and *N. armatus*'s guts respectively (Fig. 4 and 5). Fifty percent of all unique prey OTUs were identifiable to the species level while an additional 29.7% was identifiable to a higher taxonomic level. Only 20.3% of recovered dietary items could not be confidently assigned to any taxonomic level. A representative sequence of each OTU was deposited in GenBank (accession numbers JF905638 to JF905693).

In terms of the diversity of prey recovered from *P. arcatus* gut contents, there was a significant interaction between removal strategy and primer set (Repeated-measures ANOVA: *F_ 1,5_  = 6.81; p* = 0.048). There was no significant effect of the primer set on prey diversity when using the restriction enzyme (paired *t*-test: *t_5_*  = 1.50, *p* = 0.195), but we were able to detect a higher mean diversity of prey with the “COI” primer set than the “dgCOI” primer set when using the blocking primer (*t_5_*  = 3.40, *p* = 0.009). The mean number of prey species amplified using the blocking primer was significantly higher than with the restriction enzymes regardless of the primer set used (“COI”, *t_5_*  = 5.07, *p* = 0.002; “dgCOI”: *t_5_*  = 2.77, *p* = 0.019). In terms of *N. armatus*, a significantly higher mean number of prey was recovered using the blocking primer compared to restriction enzymes (“dgCOI”: *t_5_*  = 3.28, *p*  =  0.011). We found no correlation between the number of prey OTUs and the proportion of bacterial sequences (in clone libraries) recovered from hawkfish gut contents (Figure S1 and Table S2).

The majority of the prey OTUs obtained after predator DNA restriction (95%, 37 out of 39) were also recovered using the predator DNA blocking method. Most of the prey taxa which were amplified using the blocking primers 91% (49 out of 54) displayed at least one mismatch within the first 4 bp of the 3′end of the blocking primer. Other prey OTUs had between 3 and 6 mismatches with the blocking primer between the sites 644 and 658 of the 3′ end COI region (Fig. 4 and 5). None of the prey that were detected using blocking primers but not with restriction enzymes, were prevalent in clone libraries, except for *T. tigrina* and *P. integrirostris* obtained from *P. arcatus* guts (18% and 10.5% of sequences respectively). Neither polychaetes, nor the coral *Pocillopora*, were amplified using the “COI” primer set (Fig. 4 and 5).

## Discussion

A wide taxonomic range of prey items can be detected from semi-digested prey homogenates in predator gut contents. Versatile PCR primers may be used without additional modification for PCR amplification of prey upon the condition that predator DNA co-amplification does not prevent prey recovery. However, whenever significant predator DNA co-amplification occurs, the predator DNA removal strategy should also minimize the exclusion of prey templates. In this study, we propose methodological guidelines for excluding predator DNA when using versatile COI primers for diet samples while minimizing prey DNA cleavage or blocking (false negatives). Comparative results from dietary analyses highlight the higher efficiency of blocking primers versus restriction enzymes for prey diversity recovery from the gut contents for our two generalist predators (Fig. 4 and 5). Half of the total number of prey OTUs obtained for both species could be confidently assigned to a reference DNA barcode. Species-level prey identification provide detailed information on hawkfish feeding ecology.

### Morphological and DNA based identification of undigested and indigestible prey remains

Predator dietary analysis has traditionally involved the morphological and DNA based identification of hard remains [9], [48]–[50]. However, prey detection using these techniques is biased towards food items resistant to digestion. In order to minimize this error, sampling predators immediately after peak feeding or at regular intervals over 24 hours has been suggested to maximize chances of identifying fast degrading species [48]. Hawkfish specimens were all collected after sunset, a period during which they have been observed to actively feed. In spite of this precaution, few hard remains were retrieved from hawkfish gut contents. This is consistent with a study by Kane et al. (2009) [27] which estimated that the total number of prey attacks made by *P. arcatus* adults in their natural environment was very low (mean  = 0.06 prey attacks/fish/15 minutes). No behavioral information is available for *N. armatus*. DNA sequencing was successful for all undigested remains found in fish stomachs and provided species-level prey identification. On the other hand, no sequence information could be obtained from DNA barcoding of prey living in the mollusk shells found at the terminal section of fish intestines (neither the bivalve Pectinidae nor the gastropod Rissoidae). Tissue is progressively digested and DNA broken down into smaller pieces [51], [52] as prey transit through the digestive tract making individual DNA isolation and amplification more challenging.

Most of these undigested prey species were also found in the clone libraries constructed from PCR amplification of the total extraction of semi-digested tissue homogenate (Fig. 4 and 5) demonstrating that some of their tissue already had been partially digested. We found sequences of a hermit crab (superfamily Paguroidea) that might have been occupying the gastropod shell found in the intestines. However, no bivalve to match the bivalve shell was detected, highlighting either high DNA degradation or the potential for primer incompatibility. Overall, these results suggest that individual DNA barcoding may be unnecessary. Nonetheless, it is possible that these undigested prey, particularly the most recently consumed found in fish stomachs, would become largely dominant in clone libraries if their DNA was mixed with the most degraded DNA from the prey homogenate. Therefore, we recommend removing large undigested prey items from stomach contents analysis until further studies determine if they might prevent the detection of more digested prey.

### Performance of predator removal strategies for the detection of prey templates from semi-digested prey homogenates

Both versatile primer sets (“COI” and “dgCOI”) were designed for invertebrates [31], [32]. However, our results show that they were preferentially binding and amplifying predator fish DNA rather than invertebrate prey DNA in gut samples when no predator DNA removal technique was used. This might be due to multiple factors. Although not directly measured, predator DNA was possibly highly prevalent in hawkfish gut content DNA extracts either because of numerous epithelial cells from the digestive track or because inhibitors (i.e. gastropod mucus) prevented effective invertebrate prey DNA extraction. In addition, the large size of the target COI fragments might have favored the co-amplification of high quality (non-digested) DNA [51], [53] compared to the more sheared prey DNA. On the other hand, the “COI” primer set failed to amplify the barcoding fragment for the predator *N. armatus*. This simplified the molecular analysis because no predator DNA removal was required but it mostly highlights the limited versatility of this primer set (as discussed below).

Our study confirms that both predator DNA removal approaches, restriction enzyme and annealing blocking primer, are efficient at removing predator DNA from clone libraries [18], [20], [26]. Nevertheless, blocking primers offered more efficient prey detection for both predator species regardless of the primer set used. This is likely to be driven by two factors. First, prey can be directly excluded during restriction digestion. Despite using restriction enzymes with the lowest cutting frequency among potential prey, two prey species that have the restriction site in their COI sequence were likely removed from the dietary contents of each predator species (Fig. 4 and 5). Second, the amount of predator DNA competing for primers annealing with rare prey templates during the early cycles of the PCR reaction may decrease the efficiency of prey detection. Whereas blocking primers are able to immediately inhibit predator DNA amplification during PCR, restriction digestion prior to PCR amplification does not completely remove predator DNA. Dunshea (2009) [26] examined the efficiency of restriction digestion of genomic DNA extracted from scats at removing predator amplicons and showed that between 21–50% of predator sequences still remained in clone libraries and estimated that post-PCR restriction further reduced the proportion of predator sequences by 2–37%. We observed three distinct bands on agarose gels after the restriction digestion of all PCR products. Band sizes (∼300 bp and ∼400 bp for both *N. armatus* and *P. arcatus*) were consistent with the position of the restriction site in the COI sequence of predator species, a strong indication that a significant amount of predator DNA had not been restricted prior to PCR. The low efficiency of the pre-PCR digestion may have been detrimental for prey DNA detection. In addition, most prey OTUs (37 out of 39) detected using restriction enzymes were also recovered using blocking primers providing evidence that blocking primers were effective, as designed, at minimizing prey exclusion.

There was a significant interaction between primer set and predator DNA removal technique for prey detection from *P. arcatus* gut contents. While we detected higher mean prey diversity using the “COI” primer set compared to the “dgCOI” when used in combination with blocking primers, there was no significant difference in prey diversity between primer sets when used in combination with restriction enzymes. This is likely because low prey detection efficiency using restriction enzymes obscures the difference in amplification efficiency between primer sets.

Overall, these experimental results suggest that (1) annealing blocking primers should be preferred over restriction enzymes, and (2) the blocking primer binding site chosen in this study after meticulous examination of primer-prey mismatches could be confidently used for future predator specific blocking primer design.

### Accounting for primer bias in prey detection

Broad-range COI primers designed by Folmer et al. (1994) [31] remain commonly used for barcoding species in all metazoan invertebrate phyla. Despite their presumed versatility, amplification is in fact challenging for certain taxonomic groups such as gastropods [32] and echinoderms [54]. Meyer (2003) [32] designed degenerate primers modified from Folmer et al (1994) [31] which helped solve primer-template incompatibility issues. We show that the “COI” primer set [31] obviously missed important items such as the presence of polychaetes and coral DNA that were detected using the “dgCOI” primer set. On the other hand, primer degeneracy favors the co-amplification of bacterial genes in hawkfish gut contents but it does not seem to influence the recovery of prey items (Figure S1 and Table S2). Overall, there are significant biases in prey amplification between primer sets and we recommend using both primer sets to enhance the efficiency of dietary analysis of generalist predators targeting the COI gene.

### Taxonomic identification of prey gives preliminary insights into the feeding ecology of hawkfish

Sequence dissimilarity thresholds ranging from 5% to 10% identified a total of 24 and 32 prey species in the gut contents of *P. arcatus* and *N. armatus* respectively. This threshold is in accordance with previous studies targeting COI which used 5% for species delineation [35]–[37]. Species level matches with BIOCODE reference specimens were achieved for larger prey species (macrobiota) collected and identified at their adult benthic stage, whereas small bodied taxonomic groups such as copepods and amphipods are less represented in the database.

Rarefaction curves indicate that additional specimens should be collected to better characterize the diet of these hawkfish species (Figure S2). Nevertheless, species-level prey identification provided interesting new information on the feeding strategies of these fish. For example, the molecular analyses of gut contents revealed unexpected prey items given our knowledge of the feeding behavior of these predatory species. *Paracirrhites arcatus* are known to ambush large benthic prey but they were found to consume *Paracalanus parvus*, a pelagic copepod species, which potentially suggests an additional or alternative feeding strategy. Moreover, both fish species had traces of the DNA of *Pocillopora* coral in their guts. If they actively consume coral tissue, this would be the first record of corallivory in the family Cirrhitidae. On the other hand, coral DNA might be detectable in fish guts as a result of secondary predation (hyperpredation) [15], [55], [56]. This would occur if a prey captured had previously fed upon coral tissue. We also found evidence of predation upon *Trapezia tigrina*, a decapod species known to have a mutualistic relationship with *Pocillopora* corals. Twelve species of coral crabs (genus *Trapezia*) are known to live among the branches of *Pocillopora* in Moorea in close proximity with hawkfish predators [57]. These species promote the survival and growth of their host by defending against corallivorous seastars [58], removing sediments off the coral tissue [59], [60] and decreasing the negative effects of vermetid snail nets [61]. Therefore, predation might have negative effects on corals. Additional molecular analyses of hawkfish gut contents, behavioral observations and field experiments are ongoing to better comprehend the role of these predatory fish on the coral-crab mutualism.

### The future of COI in molecular analyses of predation

The ability to accurately identify detected prey by matching unknown sequences to sequences of described taxa will also determine the quality of dietary analyses. The choice of the target gene will therefore be dependent upon both the availability of databases for matching with potential prey consumed, but also upon the level of taxonomic resolution required for prey characterization. The COI gene enables species level discrimination for most metazoan groups and ever since it was proposed as an appropriate ‘taxon barcode’ for animals [62], large COI sequence libraries have become available for ecologists [63], [64].

In order to maximize the chances of prey identification, the COI gene is likely to become a preferential target in most ecological studies. However, the length of COI amplicons (658bp) is problematic for its routine use in dietary analysis for two reasons. As large DNA strands break down more quickly with digestion [65], [66] the success of prey detection will potentially be higher by targeting small DNA fragments (<300 bp) [66]. The barcoding region of the COI coding gene possesses a high level of variability among taxa causing difficulties in the design of internal primer sets binding to flanking regions conserved across a wide range of taxa [22], [52]. The mini-barcode primer set represents one attempt at designing versatile primers to target a short (∼150 bp) fragment of the 658 bp barcoding region [67]. Yet, large numbers of mismatches in the priming sites affect its efficiency across a broad range of taxa [68]. We emphasize the need for designing versatile primers targeting shorter COI fragments for its routine use in dietary analysis of generalist diets coupled with high throughput sequencing to provide novel and quick insights into fundamental ecological processes in marine and terrestrial ecosystems.

## Supporting Information

Figure S1 **Effect of bacteria co-amplification on the number of prey Operational Taxonomic Units (OTUs) recovered from fish gut contents.** Symbols and colors represent COI primer set (“COI” – square; “dgCOI” – triangle) and predator species (*Neocirrhites armatus* – red; *Paracirrhites arcatus* – blue).
(TIF)

Figure S2 **Sample based rarefaction curves for the number of prey species as a function of the number of samples.** Samples represent clone libraries obtained from fish gut contents. Lower and upper lines represent 95% CI. Photo credit: Thomas Vignaud.
(TIF)

Table S1 **Cutting frequency of commercially available restriction enzymes across ≈330 decapod taxa (947 sequences), ≈430 ray-finned fish taxa (758 sequences) and ≈170 gastropod taxa (271 sequences).**
(DOCX)

Table S2 **Test for the correlation between the % of bacterial sequence in clone libraries and the number of prey OTUs.**
(DOCX)
