# Supplementary figures and images for: Effectiveness of Annealing Blocking Primers versus Restriction Enzymes for Characterization of Generalist Diets: Unexpected Prey Revealed in the Gut Contents of Two Coral Reef Fish Species
Source: PLoS One. 2013 Apr 8;8(4):e58076. doi: 10.1371/journal.pone.0058076 (PMC3620324; doi:10.1371/journal.pone.0058076)

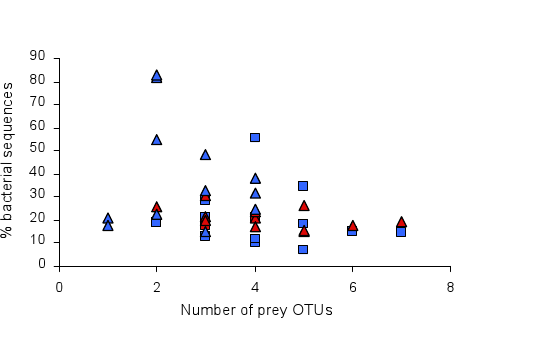

Supplement: Figure S1 — Effect of bacteria co-amplification on the number of prey Operational Taxonomic Units (OTUs) recovered from fish gut contents. Symbols and colors represent COI primer set (“COI” – square; “dgCOI” – triangle) and predator species (Neocirrhites armatus – red; Paracirrhites arcatus – blue). (TIF) [file pone.0058076.s001.tif]

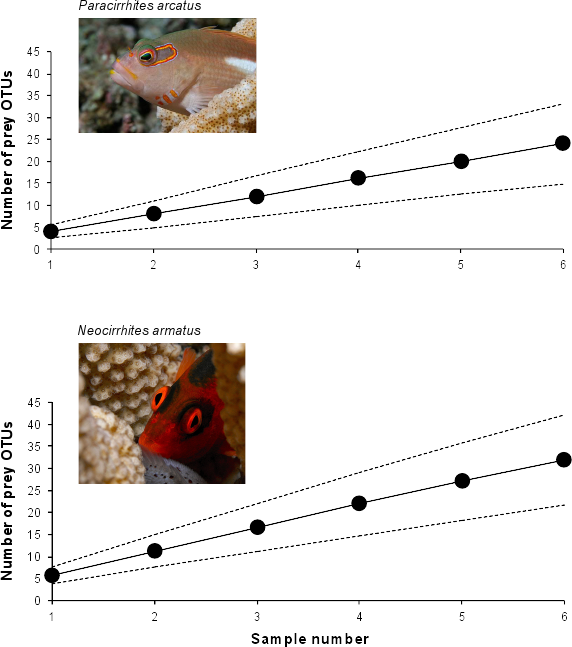

Supplement: Figure S2 — Sample based rarefaction curves for the number of prey species as a function of the number of samples. Samples represent clone libraries obtained from fish gut contents. Lower and upper lines represent 95% CI. Photo credit: Thomas Vignaud. (TIF) [file pone.0058076.s002.tif]
