# Supplementary material for: Effectiveness of Annealing Blocking Primers versus Restriction Enzymes for Characterization of Generalist Diets: Unexpected Prey Revealed in the Gut Contents of Two Coral Reef Fish Species
Source: PLoS One. 2013 Apr 8;8(4):e58076. doi: 10.1371/journal.pone.0058076 (PMC3620324; doi:10.1371/journal.pone.0058076)
Supplement: Table S1 — Cutting frequency of commercially available restriction enzymes across ≈330 decapod taxa (947 sequences), ≈430 ray-finned fish taxa (758 sequences) and ≈170 gastropod taxa (271 sequences). (DOCX) [file pone.0058076.s003.docx]

| Endonuclease | Isoschizomers | Site 5'-3' | Decapod (%) | Fish (%) | Gastropod (%) |
| --- | --- | --- | --- | --- | --- |
| AcyI | BsaHI, BstACI, Hin1I, Hsp92I | GRCGYC | 8 | 18 | 4 |
| AfeI | Aor51HI, Eco47III, FunI | AGCGCT | 0 | 1 | 2 |
| AflII | BfrI, BspTI, Bst98I, MspCI, Vha464I | CTTAAG | 8 | 14 | 5 |
| AflIII |  | ACRYGT | 4 | 5 | 7 |
| AgeI | AsiGI, BshTI, CspAI, PinAI | ACCGGT | 4 | 3 | 5 |
| AhlI | BcuI, SpeI | ACTAGT | 26 | 5 | 12 |
| Alw21I | AspHI, Bbv12L, BsiHKAI | CWGCWC | 48 | 23 | 57 |
| Alw44I | ApaLI, VneI | GTGCAC | 4 | 1 | 3 |
| Ama87I | AvaI, BsiHKCI, BsoBI, Eco88I, NLi3877I, NspIII | CYCGRG | 5 | 19 | 8 |
| Aor13HI | BIfI, BseAI, Bsp13I, BspEI, Kpn2I, MroI | TCCGGA | 2 | 9 | 7 |
| ApaI | Bsp120I, PspOMI | GGGCCC | 7 | 24 | 8 |
| ApoI | XapI | RAATTY | 0 | 0 | 0 |
| AseI | PshBI, VspI | ATTAAT | 72 | 58 | 69 |
| Asp718I | KpnI | GGTACC | 9 | 6 | 13 |
| AspA2I | AvrII, BInI, XmaJI | CCTAGG | 10 | 18 | 4 |
| AssI | ScaI, ZrmI | AGTACT | 4 | 21 | 5 |
| AsuII | Bpu14I, Bsp119I, BspT104I, BstBI, Csp45I, NspV, sfuI | TTCGAA | 2 | 0 | 2 |
| AsuNHI | BmtI, NheI | GCTAGC | 4 | 8 | 2 |
| AviII | FspI, NsbI | TGCGCA | 3 | 1 | 3 |
| BalI | MIsI, MIuNI, MscI, Msp20I | TGGCCA | 4 | 0 | 2 |
| BamHI |  | GGATCC | 9 | 5 | 18 |
| BanI | BshNI, BspT107I | GGYRCC | 39 | 44 | 36 |
| BanII | Eco24I, EcoT38I, FriOI | GRGCYC | 61 | 62 | 63 |
| BanIII | Bsa29I, BseCI, Bsp106I, BspDI, BspXI, Bsu15I, BsuTUI, ClaI, ZhoI | ATCGAT | 2 | 2 | 4 |
| BauI | BssSI, Bst2BI | CACGAG | 0 | 1 | 0 |
| BauI+ | BssSI+, Bst2BI+ | CTCGTG | 0 | 1 | 1 |
| BbeI | EgeI, EheI, KasI, Mly113I, NarI, SfoI | GGCGCC | 6 | 14 | 2 |
| BbrPI | Eco72I, PmaCI, PmII, PspCI | CACGTG | 0 | 0 | 0 |
| BbuI | PaeI, SpaHI, SphI | GCATGC | 5 | 9 | 13 |
| BclI | FbaI, Ksp22I | TGATCA | 14 | 3 | 33 |
| BfmI | BpcI, BstSFI, SfcI | CTRYAG | 60 | 34 | 55 |
| BfrBI | EcoT22I, Mph1103I, NsiI, Ppu10I, Zsp2I | ATGCAT | 8 | 20 | 10 |
| BglII |  | AGATCT | 11 | 21 | 12 |
| Bme1580I | BseSI | GKGCMC | 24 | 49 | 22 |
| BmgBI | BtrI | CACGTC | 1 | 0 | 4 |
| BmgBI+ | BtrI+ | GACGTG | 0 | 0 | 5 |
| BmyI | Bsp1286I, MhII, SduI | GDGCHC | 93 | 86 | 93 |
| BsaAI | BstBAI | YACGTR | 0 | 0 | 0 |
| BsaJI | BseDI, BssECI | CCNNGG | 46 | 75 | 53 |
| BsaMI+ | BsmI+, Mva1269I+, PctI+ | GCATTC | 17 | 50 | 8 |
| BsaWI |  | WCCGGW | 0 | 0 | 0 |
| Bse118I | BsrFI, BssAI, Cfr10I | RCCGGY | 0 | 0 | 0 |
| Bse3DI+ | BseMI+, BsrDI+ | CATTGC | 7 | 0 | 5 |
| BsePI | BssHII, PauI | GCGCGC | 0 | 1 | 0 |
| BseX3I | BstZI, EagI, EcIXI, Eco52I | CGGCCG | 2 | 6 | 2 |
| BseYI |  | CCCAGC | 1 | 36 | 1 |
| BseYI+ |  | GCTGGG | 22 | 18 | 30 |
| Bsh1285I | BsiEI, BstMCI | CGRYCG | 16 | 18 | 14 |
| BsiWI | PfI23II, PspLI, SunI | CGTACG | 0 | 0 | 0 |
| Bsp1407I | BsrGI, BstAUI, SspBI | TGTACA | 0 | 0 | 2 |
| Bsp143II | BstH2I, HaeII, LpnI | RGCGCY | 0 | 0 | 0 |
| Bsp19I | NcoI | CCATGG | 2 | 0 | 4 |
| Bsp68I | NruI | TCGCGA | 0 | 0 | 0 |
| BspCI | MvrI, Ple19I, PvuI | CGATCG | 8 | 6 | 8 |
| BspHI | PagI, RcaI | TCATGA | 6 | 28 | 2 |
| BspLI | NIaIV, PspN4I | GGNNCC | 86 | 96 | 86 |
| BspLU11I | PciI, PscI | ACATGT | 0 | 0 | 0 |
| BspMAI | PstI | CTGCAG | 7 | 23 | 25 |
| BsrBI | MbiI | CCGCTC | 6 | 7 | 7 |
| BsrBI+ | MbiI+ | GAGCGG | 5 | 8 | 16 |
| BssHI | PaeR7I, SciI, Sfr274I, SIaI, StrI, TIiI, XhoI | CTCGAG | 1 | 0 | 0 |
| BssNAI | Bst1107I, BstZ17I | GTATAC | 22 | 12 | 16 |
| BssT1I | Eco130I, EcoT14I, ErhI, StyI | CCWWGG | 14 | 34 | 13 |
| BstC8I | Cac8I | GCNNGC | 33 | 100 | 32 |
| BstDSI | BtgI | CCRYGG | 6 | 8 | 12 |
| BstNSI | NspI, XceI | RCATGY | 0 | 0 | 0 |
| BstSNI | Eco1005I, SnaBI | TACGTA | 0 | 0 | 1 |
| BstX2I | BstYI, MfII, PsuI, XhoII | RGATCY | 0 | 0 | 0 |
| BtsI+ |  | CACTGC | 11 | 26 | 10 |
| Cfr42I | KspI, SacII, Sfr303I, SgrBI | CCGCGG | 2 | 3 | 4 |
| Cfr9I | PspAI, SmaI, UthSI, XmaCI, XmaI | CCCGGG | 2 | 11 | 4 |
| CfrI | EaeI | YGGCCR | 0 | 0 | 0 |
| DraI |  | TTTAAA | 33 | 9 | 14 |
| Ecl136II | EcoICRI, Psp124BI, SacI, SstI | GAGCTC | 26 | 12 | 32 |
| Eco147I | PceI, SseBI, StuI | AGGCCT | 2 | 0 | 4 |
| Eco32I | EcoRV | GATATC | 0 | 0 | 1 |
| EcoRI | FunII | GAATTC | 1 | 0 | 2 |
| FauNDI | NdeI | CATATG | 1 | 0 | 1 |
| FblI | XmiI | GTMKAC | 47 | 63 | 36 |
| GdiII |  | CGGCCR | 6 | 9 | 5 |
| GdiII+ |  | YGGCCG | 0 | 0 | 0 |
| HaeI |  | WGGCCW | 0 | 0 | 0 |
| HinCII | HindII | GTYRAC | 55 | 48 | 44 |
| HindIII |  | AAGCTT | 1 | 44 | 15 |
| HpaI | KspAI | GTTAAC | 16 | 2 | 18 |
| Hpy188III |  | TCNNGA | 66 | 64 | 81 |
| Hpy8I |  | GTNNAC | 91 | 93 | 82 |
| MfeI | MunI | GAATTG | 9 | 2 | 10 |
| MluI |  | ACGCGT | 2 | 4 | 5 |
| MroNI | NaeI, NgoMIV, PdiI | GCCGGC | 5 | 25 | 0 |
| MspA1I |  | CMGCKG | 23 | 36 | 48 |
| PsiI |  | TTATAA | 96 | 60 | 70 |
| Psp1406I |  | AACGTT | 9 | 7 | 7 |
| PvuII |  | CAGCTG | 13 | 18 | 32 |
| SalI |  | GTCGAC | 4 | 10 | 4 |
| SmlI |  | CTYRAG | 14 | 16 | 13 |
| SspI |  | AATATT | 34 | 2 | 25 |
| TatI |  | WGTACW | 0 | 0 | 0 |
| XbaI |  | TCTAGA | 9 | 0 | 3 |
| ZraI |  | GACGTC | 0 | 0 | 1 |
| AxyI | Bse21I, Bsu36I, Eco81I | CCTNAGG | 5 | 9 | 1 |
| BbvCI |  | CCTCAGC | 1 | 3 | 4 |
| BbvCI+ |  | GCTGAGG | 0 | 0 | 0 |
| BlpI | Bpu1102I, Bsp1720I, CeIII | GCTNAGC | 6 | 27 | 7 |
| Bpu10I |  | CCTNAGC | 5 | 36 | 9 |
| Bpu10I+ |  | GCTNAGG | 0 | 0 | 1 |
| BstEII | BstPI, Eco91I, EcoO65I, PspEI | GGTNACC | 10 | 54 | 13 |
| CpoI | CspI, Rsr2I, RsrII | CGGWCCG | 2 | 9 | 4 |
| DraII | EcoO109I, PssI | RGGNCCY | 0 | 0 | 0 |
| MabI | SexAI | ACCWGGT | 23 | 5 | 9 |
| PasI |  | CCCWGGG | 2 | 0 | 2 |
| PfoI |  | TCCNGGA | 1 | 1 | 2 |
| PpuMI | PpuXI, Psp5II, PspPPI | RGGWCCY | 0 | 0 | 0 |
| SanDI |  | GGGWCCC | 11 | 14 | 10 |
| Sse8647I |  | AGGWCCT | 1 | 0 | 3 |
| AscI | PaIAI, SgsI | GGCGCGCC | 0 | 1 | 0 |
| AsiSI | SgfI | GCGATCGC | 0 | 0 | 0 |
| CciNI | NotI | GCGGCCGC | 1 | 0 | 0 |
| FseI |  | GGCCGGCC | 0 | 0 | 0 |
| FspAI |  | RTGCGCAY | 0 | 0 | 0 |
| MssI | PmI | GTTTAAAC | 0 | 0 | 0 |
| PacI |  | TTAATTAA | 0 | 0 | 0 |
| PspXI |  | VCTCGAGB | 0 | 0 | 0 |
| SbfI | SdaI, Sse8387I | CCTGCAGG | 0 | 17 | 0 |
| SgrAI |  | CRCCGGYG | 2 | 2 | 5 |
| SmiI | SwaI | ATTTAAAT | 22 | 3 | 1 |
| SrfI |  | GCCCGGGC | 0 | 0 | 0 |
| Sse232I |  | CGCCGGCG | 1 | 1 | 0 |
| AdeI | DraIII | CACNNNGTG | 0 | 2 | 1 |
| AlwNI | CaiI | CAGNNNCTG | 3 | 11 | 23 |
| AspI | PfIFI, PsyI, TeII, Tth111I | GACNNNGTC | 6 | 0 | 2 |
| AleI | OliI | CACNNNNGTG | 8 | 11 | 9 |
| Asp700I | MroXI, PdmI, XmnI | GAANNNNTTC | 30 | 4 | 18 |
| BoxI | BstPAI, PshAI | GACNNNNGTC | 1 | 0 | 0 |
| BsaBI | Bse8I, BseJI, MamI | GATNNNNATC | 11 | 11 | 5 |
| MslI | SmiMI | CAYNNNNRTG | 34 | 44 | 39 |
| AhdI | AspEI, DriI, Eam1105I, EcIHKI | GACNNNNNGTC | 2 | 0 | 0 |
| ApaBI | BstAPI | GCANNNNNTGC | 16 | 10 | 3 |
| BglI |  | GCCNNNNNGGC | 5 | 44 | 4 |
| Bsc4I | BseLI, BsiYI, BsII | CCNNNNNNNGG | 55 | 100 | 93 |
| BstENI | EcoNI, XagI | CCTNNNNNAGG | 3 | 40 | 18 |
| BstMWI | HpyF10VI, MwoI | GCNNNNNNNGC | 86 | 98 | 46 |
| PflBI | PfIMI, Van91I | CCANNNNNTGG | 0 | 7 | 4 |
| BstXI |  | CCANNNNNNTGG | 4 | 2 | 8 |
| DrdI | DseDI | GACNNNNNNGTC | 0 | 0 | 1 |
| SfiI |  | GGCCNNNNNGGCC | 0 | 2 | 1 |
| XcmI |  | CCANNNNNNNNNTGG | 4 | 3 | 7 |
