# Supplementary material for: Effectiveness of Annealing Blocking Primers versus Restriction Enzymes for Characterization of Generalist Diets: Unexpected Prey Revealed in the Gut Contents of Two Coral Reef Fish Species
Source: PLoS One. 2013 Apr 8;8(4):e58076. doi: 10.1371/journal.pone.0058076 (PMC3620324; doi:10.1371/journal.pone.0058076)
Supplement: Table S2 — Test for the correlation between the % of bacterial sequence in clone libraries and the number of prey OTUs. (DOCX) [file pone.0058076.s004.docx]

| Predator species | DNA removal | Primer set | n | Pearson coefficient | *P* value |
| --- | --- | --- | --- | --- | --- |
| *Neocirrhites armatus* | none | COI | 6 | 0.58 | 0.23 |
|  | Enzyme | dgCOI | 6 | 0.16 | 0.76 |
|  | Blocking | dgCOI | 6 | 0.05 | 0.93 |
| *Paracirrhites arcatus* | Enzyme | COI | 6 | 0.42 | 0.4 |
|  | Enzyme | dgCOI | 6 | 0.08 | 0.88 |
|  | Blocking | COI | 6 | 0.77 | 0.07 |
|  | Blocking | dgCOI | 6 | 0.47 | 0.35 |
